# Resolution of Pulsatile Tinnitus after Venous Sinus Stenting in Patients with Idiopathic Intracranial Hypertension

**DOI:** 10.1371/journal.pone.0164466

**Published:** 2016-10-21

**Authors:** Srikanth Boddu, Marc Dinkin, Maria Suurna, Kelly Hannsgen, Xem Bui, Athos Patsalides

**Affiliations:** 1 Department of Neurological surgery, Division of Interventional Neuroradiology, New York Presbyterian Hospital / Weill Cornell Medical Center, New York, New York, United States of America; 2 Department of Ophthalmology, New York Presbyterian Hospital / Weill Cornell Medical Center, New York, New York, United States of America; 3 Department of Otolaryngology, New York Presbyterian Hospital / Weill Cornell Medical Center, New York, New York, United States of America; University of Texas at Dallas, UNITED STATES

## Abstract

**Objective:**

Evaluate the role of venous sinus stenting in the treatment of pulsatile tinnitus among patients with Idiopathic Intracranial Hypertension (IIH) and significant venous sinus stenosis.

**Subjects and Methods:**

A written informed consent approved by the Weill Cornell institutional review board was signed and obtained from the study participants. Thirty-seven consecutive patients with IIH and venous sinus stenosis who were treated with venous sinus stenting between Jan.2012-Jan.2016 were prospectively evaluated. Patients without pulsatile tinnitus were excluded. Tinnitus severity was categorized based on “Tinnitus Handicap Inventory” (THI) at pre-stent, day-0, 1-month, 3-month, 6-month, 12-month, 18-month and 2-year follow-up. Demographics, body-mass index (BMI), pre and post VSS trans-stenotic pressure gradient were documented. Statistical analysis performed using Pearson’s correlation, Chi-square analysis and Fischer’s exact test.

**Results:**

29 patients with a mean age of 29.5±8.5 years M:F = 1:28. Median (mean) THI pre and post stenting were: 4 (3.7) and 1 (1) respectively. Median time of tinnitus resolution post VSS was 0-days. There was significant improvement of THI (Δ Mean: 2.7 THI [95% CI: 2.3–3.1 THI], p<0.001) and transverse-distal sigmoid sinus gradient (Δ Mean: -15.3 mm Hg [95% CI: 12.7–18 mm Hg], p<0.001) post-stenting. Mean follow-up duration of 26.4±9.8 months (3–44 months). VSS was feasible in 100% patients with no procedural complications. Three-patients (10%) had recurrent sinus stenosis and tinnitus at mean follow-up of 12 months (6–30 months).

**Conclusion:**

Venous sinus stenting is an effective treatment for pulsatile tinnitus in patients with IIH and venous sinus stenosis.

## Introduction

Pulsatile tinnitus (PT) is described as a conscious and undesired perception of heartbeat in the ear of affected individuals. Pulsatile tinnitus can be classified by its site of generation as arterial, arteriovenous, or venous. PT not only reflects the pulse-synchronous sounds of vascular origin but also the rhythmic sounds which are not pulse-synchronous and which are related to other sources like muscular contractions (e.g. stapedius muscle). Pulsatile tinnitus can have many causes. Typical arterial causes are arteriosclerosis, dissection, and fibromuscular dysplasia. Common causes at the arteriovenous junction include arteriovenous fistulae and highly vascularized skull base tumors. Common venous causes are intracranial hypertension and, as predisposing factors, anomalies and normal variants of the basal veins and sinuses. Idiopathic Intracranial Hypertension (IIH), also known as pseudotumor cerebri, is by far the most common cause of pulsatile tinnitus in young and obese female patients[1]. The original criteria for diagnosis of IIH was described by Dandy in 1937[2] and a modified by Smith in 1985 to become “modified Dandy criteria” replacing ventriculography with computed tomography (CT) for imaging[3]. This was further amended by Digre and Corbett in 2001, included awake and alert patient and exclusion of venous sinus thrombosis in the diagnostic criteria[4].

IIH is a condition seen in obese women of childbearing age. Although the incidence is 1 in 100,000 in normal-weight individuals, the incidence jumps to 20 in 100,000 in women who are obese[4]. Headache and/or visual disturbance are the usual manifestation of IIH syndrome. Pulsatile tinnitus as an initial presentation of IIH syndrome was first reported in 1985[5]. Persistent nature of pulsatile tinnitus can significantly affect patients' sleep and quality of life, leading to depression in severe cases[6].

Russell’s et al. first reported the association between venous sinus stenosis and tinnitus[7] in 1995. Two-years later Mathis et al. first reported a case of intracranial hypertension and venous sinus stenosis, in which refractory pulsatile tinnitus resolved after venous sinus stenting (VSS)[8]. Since then, there is increasing awareness of venous sinus stenosis as a potential etiology of pulsatile tinnitus.

Farb et al.[9] have identified the presence of venous sinus stenosis in more than 90% of patients with IIH, compared to a mere 6.8% in the control asymptomatic group. Riggeal et al.[10] reported bilateral transverse sinus stenosis in 90% of their IIH cohort. However, the exact role of the venous sinus stenosis in IIH is a debatable topic. Studies reporting the normalization of stenosis after CSF drainage with lumbar puncture or shunting procedures[11] support venous stenosis as a consequence of IIH. In contrary, studies reporting persistence of stenosis in spite of CSF drainage consider sinus stenosis as an etiology of IIH[12]. Lateral (transverse and sigmoid) sinus stenosis is a common pathology in IIH, disrupting the normal blood flow from a stenotic segment into a distal dilation resulting in turbulence that can be transmitted to the cochlea via osseous conduction, leading to perception of pulsatile tinnitus[13].

There is limited literature regarding the impact of venous sinus stenting on pulsatile tinnitus, mostly confined as a secondary outcome in patients with IIH.[14,15] The aim of our study is quantitative evaluation venous sinus stenting in the treatment of pulsatile tinnitus among patients with IIH and significant lateral sinus stenosis. We hypothesize that venous sinus stenting across the stenosis alters venous flow dynamics, minimizes turbulence thus resolves the pulsatile tinnitus.

## Materials and Methods

### Patient Population

All patients with IIH treated venous sinus stenting at our institution over a 4-year period (January 2012-January 2016) were prospectively evaluated. A written informed consent approved by the Weill Cornell institutional review board was signed and obtained from the study participants. All patients had persistent symptoms of increased intracranial pressure. The diagnosis of IIH was based on combined clinical (refractory headaches ± visual symptoms despite maximal medical management, including carbonic anhydrase inhibitors and diuretics), imaging (venous sinus stenosis, either bilateral or unilateral in dominant sinus), documented high opening pressure on lumbar puncture and an elevated (> = 8 mm Hg) trans-stenotic gradient on venous manometry. Based on the presence of visual symptoms at presentation, the patients were either enrolled in the FDA approved prospective clinical trial of “Venous sinus stenting in patients with idiopathic intracranial hypertension refractory to medical therapy” (ClinicalTrials.gov, NCT01407809) or were enrolled in a prospective patient registry, both approved by the Institutional Review Board. Twenty-nine of the 37 IIH patients (n = 29, 78%) in our database (RedCap) had pulsatile tinnitus at initial clinical presentation and constitute our study population.

### Data Collection

All data were collected prospectively. Patient demographics, body-mass index (BMI), procedural details, complications, pre and post procedural tinnitus handicap inventory (THI) and trans-stenotic pressure gradient were obtained from the database. Data on transverse sinus symmetry, stenosis localization, pre and post preferential flow in the transverse sinuses were collected from contrast-enhanced MR venography (MRV) and catheter venography before and after stenting. The pre-, intra- and post- procedural patient care, evaluation and follow-up were identical irrespective of whether the patients were part of the trial or the registry.

#### Tinnitus severity and laterality

Patients were evaluated for pulsatile tinnitus based on clinical history and physical exam. Venous etiology of the pulsatile tinnitus was confirmed in our patients based on internal jugular vein compression. Severity of tinnitus was graded from 1–5 (1 being mild or no handicap, and 5 being catastrophic) based on THI[16]. The same questionnaire was used for assessment at pre-stent, day-0, 1-month, 3-month, 6-month, 12-month, 18-month and 2-year follow-up. Patients were routinely investigated for non-venous etiology of pulsatile tinnitus.

#### Transverse sinus symmetry and preferential flow

**Fig 1:**
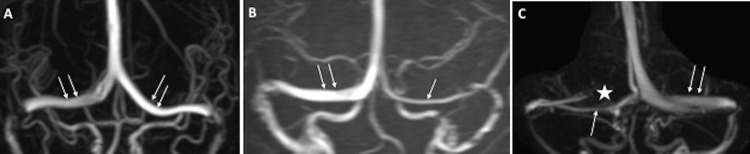
Patterns of venous sinus system dominance on MRV. A: Co-dominant sinus system (Double white arrows on each dominant transverse sinus; TS); B: Dominant-Hypoplastic sinus system (Double white arrows: Dominant TS and Single white arrow: Hypoplastic TS); C: Dominant-Aplastic sinus system (Double white arrows: Dominant TS; *: Aplastic sinus; Single white arrow: Collateral venous channel draining inferior sagittal sinus.

**Fig 2:**
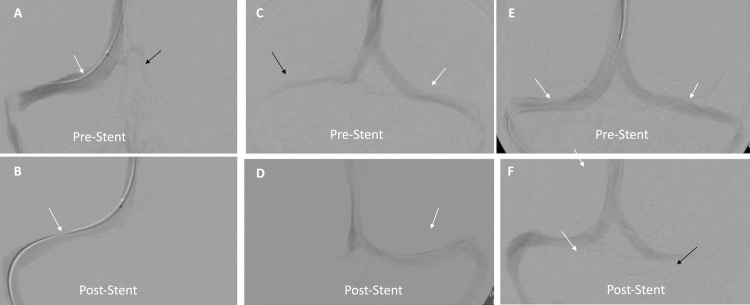
Alteration in flow dynamics following venous sinus stenting in different dominance patterns of transverse sinuses. Patient-1 (A&B): Dominant-aplastic system (white arrow) with venous collaterals (Black arrow). Post venous stent preferential flow across the unilateral dominant sinus with no opacification of collaterals. Patient-2 (C&D): Dominant (white arrow)-Hypoplastic (black arrow) system. Post venous stent preferential flow across the left dominant TS (white arrow) with non-visualization of hypoplastic TS. Patient-3 (E&F): Co-dominant sinus system (white arrows). Post venous stent of non-dominant (right) hemisphere TS shows preferential flow across right TA (white arrow) and slow flow on the contralateral side (black arrow).

Based on anatomic symmetry on contrast-enhanced MRV, transverse sinuses were categorized in to: co-dominant, dominant-hypoplastic and dominant-aplastic systems (Fig 1). Asymmetric transverse sinuses with a difference of >3 mm in the maximal antero-posterior diameter of the bilateral transverse sinuses was used to differentiate hypoplastic from co-dominant transverse sinus.[17] Absence of one transverse sinus was categorized as aplastic system. Preferential sinus flow pattern in transverse sinuses was grouped under “unilateral”, “bilateral” and “bilateral with preferential flow in stented sinus” categories before and after the venous sinus stent to evaluate impact of VSS on sinus flow dynamics (Fig 2).

#### Localization of venous sinus stenosis

Focal or diffuse flow limiting narrowing of a dural venous sinus was defined as “venous sinus stenosis”. Laterality and location of the VSS was documented based on the contrast MRV. Unilateral stenosis was defined as involvement of the dominant lateral sinus. Bilateral stenosis was defined as involvement of both lateral sinuses irrespective of co-dominant or hypoplastic system. Two independent observers (AP, SB) evaluated the venous sinus symmetry and localization of the venous sinus stenosis based pre-procedural contrast–enhanced MRV, while the preferential transverse sinus flow before and after stenting was evaluated bade on catheter venography performed during the stenting procedure under general anesthesia.

#### Body-mass Index (BMI)

The BMI was calculated on the basis of the height and weight documented in the records by using the formula: Weight (kg) / [Height (m)] ^2^. BMI categorization was performed using the modified obesity guidelines of the National Heart, Lung, and Blood Institute: underweight (BMI < 18.5), normal (BMI = 18.5–24.9), overweight (BMI = 25–29.9), obese (BMI = 30–39.9), and extremely obese (BMI ≥ 40).[18]

#### Venous manometry

Prior to stenting, the patients were evaluated with venous manometry to confirm the presence and severity of stenosis. Under local anesthesia, a 2.3 French microcatheter was advanced via the femoral vein into the dural venous sinuses. Venous manometry involved measurement of the pressure at the superior sagittal sinus, proximal and distal transverse sinus, proximal and distal sigmoid sinus, and the jugular bulb. Proximity of the transverse and sigmoid sinuses is defined in respect to the torcula. The trans-stenotic pressure gradient was measured as the pressure difference on venous manometry between the proximal transverse sinus and the distal sigmoid sinus. The presence of a minimum trans-stenotic gradient of 8mm Hg was required to proceed with stenting.

#### Venous sinus stenting

Stenting was performed immediately after manometry (27/29 patients) or on a separate day (2/29). All patients were premedicated with aspirin and clopidogrel for at least 48 hours. Under general anesthesia, the stenosis was localized using intravenous ultrasound (Volcano Eagle Eye gold catheter, Volcano Corporation). Subsequently, a self-expanding stent (diameter: 8–10 mm, length: 20–60 mm) was carefully deployed in the lateral sinus across the stenosis under fluoroscopic guidance. The stent typically extended from the mid transverse to the mid sigmoid sinus. The trans-stenotic pressure gradient was measured immediately post-stenting. Only one lateral sinus was treated with stenting: the dominant sinus was chosen for in patients with unilateral dominant sinus, i.e. dominant-hypoplastic and dominant-aplastic systems. In patients with co-dominant system, the non-dominant hemisphere was chosen.

#### Follow-up

The presence and severity of after stenting tinnitus was evaluated with the THI questionnaire at day-0, 1-month, 3-month, 6-month, 12-month, 18-month and 2-year follow-up visits. Post-procedural contrast-enhanced MRV was performed at 3 and 24 months post-stenting, and at other times if clinically indicated.

### Statistical Analysis

Statistical analysis was performed with SPSS version 21(SPSS Inc., Chicago, IL, USA). Tinnitus was considered as a categorical variable and was dichotomized as “low grade tinnitus” (grades:1–2) and “high-grade tinnitus” (grades:3–5). Continuous variables like age, BMI and pre-stent trans-stenotic gradient; categorical variables such as localization of stenosis, transverse sinus symmetry and preferential flow were considered to be the potential factors affecting the tinnitus. Mean, range and SD were calculated for the continuous variables. Inter-observer agreement for categorical variables was measured using “percent agreement statistic” (alpha). Pearson’s correlation was used to find the association between the tinnitus grade and BMI and trans-stenotic pressure gradient. Chi-square analysis was used to correlate the outcome variable (tinnitus grade) against categorical variables. Fischer exact test was used to correlate pre and post stent THI scores, trans-stenotic gradients and to evaluate association between the continuous variables. The significance level for the analyses was .05.

## Results

### Patient population

The mean age of the study population was 29.5±8.5 years (16–59 years), with 28 out of 29 being female. The mean duration of follow-up in this cohort was 26.4±9.8 months (3–44 months). The prevalence of pulsatile tinnitus in our IIH cohort was significantly higher than the visual symptoms (p = 0.02) at initial presentation. In addition to pulsatile tinnitus, 100% (n = 29) of the study population had headaches and 45% (n = 13) had papilledema at initial presentation.

### BMI

Mean BMI of the patient group was 37±7.8 kg/M^2^ (19.3–52.6 kg/M^2^). The majority of the study population were obese (n = 14; 48%) and extremely obese (n = 10; 34%). Only one patient (3%) had normal BMI. BMI as a categorical variable has no significant correlation with the tinnitus severity (p = 0.44).

### Tinnitus severity and laterality

In the presence of a co-dominant dural venous system, tinnitus caused by venous sinus stenosis was exaggerated by compression of the contralateral internal jugular vein and resolved by the compression of the ipsilateral internal jugular vein. In patients with a unilateral dominant sinus, the effect of contralateral jugular venous compression is not demonstrated, but there is still resolution of tinnitus with ipsilateral jugular vein compression, a well reported phenomenon in the literature[1,17]. The THI scores before stenting were the following: 7 patients (24%) had grade-5, 13 patients (45%) had grade-4, 5 patients (17%) had grade-3, 3 patients (10%) had grade-2 and one patient (3%) had grade-1. Median (mean) THI pre and post VSS were: 4 (3.7) and 1 (1) respectively. Fifteen patients (n = 15; 52%) had unilateral tinnitus (10: right and 5: left) and 14 patients (n = 14; 48%) had bilateral tinnitus. Unilateral tinnitus is significantly associated with ipsilateral dominant sinus stenosis (ipsilateral dominant [n = 13] versus co-dominant [n = 2] system; p<0.001). Bilateral tinnitus is significantly associated with combined stenosis of the both distal transverse sinuses in a co-dominant sinus system (co-dominant [n = 10] versus unilateral dominant [n = 4] system; p<0.01). No significant correlation was demonstrated between the tinnitus severity (high versus low grade) and laterality of stenosis (p = 0.54).

### Transverse sinus symmetry and preferential flow

Unilateral dominant sinus was detected in 17 patients (59%) and co-dominant transverse sinus in 12 patients (41%). Of the unilateral dominant system, 88% (n = 15) were dominant-hypoplastic variant while only 12% (n = 2) had dominant-aplastic pattern. Following stenting, all patients demonstrated alteration in the preferential flow. Among patients with co-dominant sinus system; 83% (n = 10) had change of preferential flow pattern from “bilateral” to “bilateral with preferential flow via stented sinus” and 17% (n = 2) changed from “bilateral” to “unilateral”. Of patients with dominant-hypoplastic system; 73% (n = 11) had flow alteration from “bilateral” to “unilateral” and 27% (n = 4) transitioned from “bilateral” to “bilateral with preferential flow via stented sinus”. As expected, “unilateral” flow pattern in patients with dominant-aplastic sinus system was unchanged following VSS.

### Localization of venous sinus stenosis

All patients (n = 29; 100%) had venous sinus stenosis localized to the distal transverse / proximal sigmoid sinus junction. The stenosis was localized to bilateral distal transvers sinus of the co-dominant system in 41% (n = 12), bilateral distal transverse sinuses of the dominant-hypoplastic system in 52% (n = 15) and to unilateral dominant system of the dominant-aplastic system in 7% (n = 2). Excellent inter-observer agreeability was noted for localization of the venous sinus stenosis (93%), transverse sinus symmetry (90%) and alteration of preferential flow pattern following VSS (86%).

### VSS—Feasibility and safety

**Fig 3:**
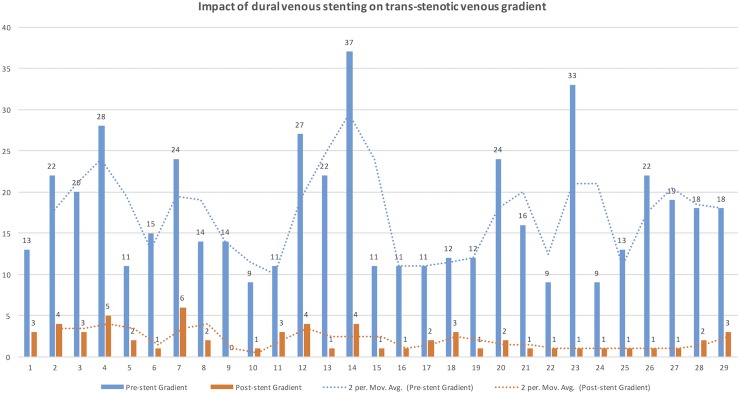
Impact of venous sinus stenting on trans-stenotic venous gradient. The trans-stenotic gradient before (blue) and after (Orange) venous stenting for each patient in the study population were plotted along the horizontal axis and trans-stenotic gradient (mm Hg) was marked along the vertical axis. Note the significant reduction of trans-stenotic pressure gradient following venous stent (p<0.001) in the entire study population.

Successful venous sinus stenting was performed in all patients, consistent with 100% technical feasibility. All patients had unilateral stenting; 76% (n = 22) in right lateral and 24% (n = 7) in the left lateral sinuses transverse-sigmoid junction. There were no neurological adverse events in our cohort. Eleven patients (38%) developed transient focal post-procedural headache at the site of stenting, treated with steroids. One patient developed a small retroperitoneal hematoma that was managed conservatively without the need for invasive treatment, blood transfusion or cessation of antiplatelet agents. Significant reduction in the trans-stenotic pressure gradient was demonstrated on the venous manometry performed immediately after venous sinus stenting (Δ Mean: -15.3 mm Hg [95% CI: 12.7–18 mm Hg], p<0.001) [Fig 3].

### Impact of VSS on tinnitus severity

**Fig 4:**
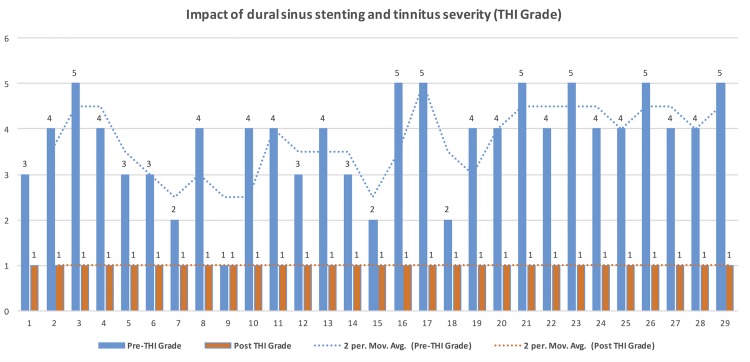
Impact of venous sinus stenting on Tinnitus severity (THI Grade). The tinnitus severity based on THI grade before (blue) and after (orange) the venous stenting for individual patients were plotted along the horizontal axis and THI severity grade was marked along the vertical axis. Complete resolution of tinnitus (grade-1) noted in all except one patient following venous stenting. One patient with grade-1(minimal) tinnitus pre-stent remained unchanged in severity following VSS (Patient-9). Please note THI grade-1 represents both mild and no tinnitus.

**Fig 5:**
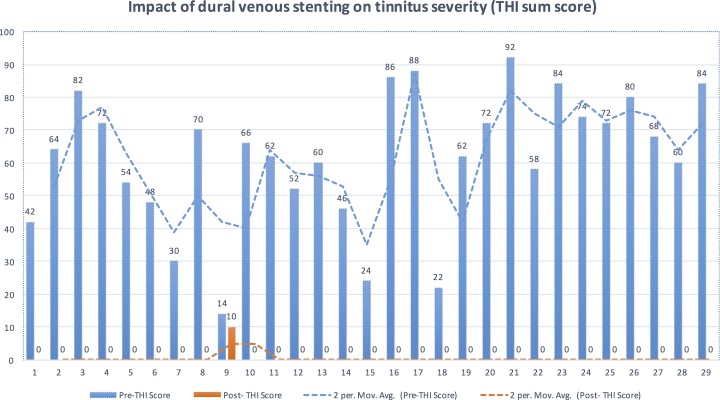
Impact of venous sinus stenting on Tinnitus severity (THI sum score). The tinnitus severity based on THI sum score before (blue) and after (orange) the venous stenting for individual patients were plotted along the horizontal axis and THI sum score was marked along the vertical axis. Complete resolution of tinnitus was noted in all except one patient following venous stenting. Evaluation based on sum score provides clear difference in the tinnitus improvement, especially in THI grade-1 patients (Patient-9).

VSS resulted in resolution of tinnitus in 28/29 patients, immediately post-procedure. One patient with grade-1 tinnitus had persistent tinnitus after the procedure, unchanged in severity. There was overall significant reduction of the tinnitus severity (Δ Mean: 2.7 THI [95% CI: 2.3–3.1 THI], p<0.001) [Figs 4 & 5]. The median duration for post stent resolution of tinnitus was 0-days i.e. same day of VSS.

### Follow-up

Except for the patients with recurrent stenosis (3/29, 10%), resolution of tinnitus persisted on 3-month, 6-month, 12-month, 18-month and 2-year follow-up. Three-patients (10%) had recurrent tinnitus caused by recurrent transverse sinus stenosis adjacent to the stent at a mean follow-up of 12 months (6–30 months). Two of these patients had a repeat stenting procedure with immediate resolution of tinnitus. The third patient opted to be treated medically. None of the patients required cerebrospinal fluid diversion procedures during follow-up.

## Discussion

Our study offers prospectively collected data that demonstrate immediate and complete resolution of pulsatile tinnitus following venous sinus stenting in patients with IIH and distal transverse sinus stenosis. The resolution of the tinnitus was appreciated by the patients on the same day of the procedure (day-0), as early as they recovered from general anesthesia. Except for the patients with recurrent stenosis (3/29, 10%), this improvement persisted on 3-month, 6-month, 12-month, 18-month and 2-year follow-up. The primary objective of treatment with venous sinus stenting in our study population i.e. patients with tinnitus is the control of refractory headaches and worsening visual symptoms from IIH. The resolution of pulsatile tinnitus following VSS served as an additional benefit in IIH patients with pulsatile tinnitus.

Even though the improvement of tinnitus following stenting for venous sinus stenosis for patients with IIH has been described in prior studies [8,15,19–21], we provide detailed information in regards to the degree and timing of improvement. More recently, Baomin et al.[13] reported similar results in 46/46 patients with venous sinus stenosis who underwent angioplasty and stenting, all patients had failed medical management. There was immediate resolution of pulsatile tinnitus in 44 patients and diminished but persistent tinnitus in 2 patients at 3-month follow-up. At 3-month follow-up, 2 patients reported recurrence of non-pulsatile tinnitus but this disappeared in both patients at the 6-month follow-up. Interestingly, only 3 of the study patients had proven association IIH, even though it’s not clear whether all patients were screened for it. In a retrospective review of 242 patients with unilateral pulsatile tinnitus, 70% of the patients had multiple vascular anomalies or variants on the symptomatic side[22], which may explain persistent low grade tinnitus in spite of treating the most obvious venous sinus stenosis in these patients.

In our experience resolution of the pulsatile tinnitus is an immediate effect appreciated by the patients upon waking up from the anesthesia, whereas improvement of the headache and visual symptoms is an intermediate effect taking at least a few days. We believe that this is related to the differences in the pathophysiological mechanism of these two symptoms. Tinnitus in these patients is a manifestation of the turbulent flow either from stenosis itself and/ or from the post-stenotic dilatation of the sigmoid sinus, transmitted to the cochlea via bone conduction[13,23]. As soon as there is no significant residual stenosis post-stenting, the blood flow normalizes and the tinnitus resolves. This notion is supported by the fact that, as in prior studies[15,19–21,24–27], we demonstrate a uniform robust reduction in the trans-stenosis gradient along with resolution of the stenosis, immediately after stent placement. On the other hand, the flow redistribution and generalized decrease in the intracranial pressure is a slow process with gradual improvement in the headaches and visual symptoms. Further evidence of direct connection between the venous sinus stenosis and pulsatile tinnitus is provided by Baomin et al[13]. where most of their patients with resolution of tinnitus post venous sinus stenting did not have signs and symptoms of increased intracranial pressure.

According to Cho et al., an abrupt change in the diameter of the lateral (transverse and sigmoid) venous sinus causes turbulent flow and is a likely mechanism of pulsatile tinnitus. Based on CT venography measurements, patients had an increased chance of pulsatile tinnitus when the smallest cross-sectional dimension was >4.75 times less than the largest cross-sectional dimension, even in an apparently normal venous system[23]. This put together with the continuity equation (**Q = AV**, where **Q** is the blood flow rate, **A** is the cross-sectional area of the vessel, and **V** is the average velocity of the blood), reduction in vessel diameter (**A**) will cause an increase in the blood flow speed (**V**). The increased flow speed results in increased turbulence, represented by the Reynold number (Re): **Re = rVL/m**, where **r** is the density of the blood, **V** is the mean velocity of the blood, **L** is the characteristic diameter of the vessel, and **m** is the dynamic viscosity of the blood. Greater turbulence may also be related to increased vibration and sound i.e. pulsatile tinnitus. In all cases with severe stenosis in the distal transverse sinus, we observed post-stenotic dilation of the sigmoid sinus, representing a diverticulum. The turbulent flow in the post-stenotic dilatation of the sigmoid sinus is transmitted to the cochlea via bone conduction. Due to increased post stenotic dilatation of the sigmoid sinus (diverticulum) there is increased apposition of the venous sinus margin to the osseous sinus wall, which facilitates the bone conduction. Following venous sinus stenting and resolution of the stenosis, there is decreased post-stenotic dilatation from the combined effect of resolution of the luminal stenosis and apposition of the sigmoid sinus wall to the venous stent. This minimizes the turbulent flow and pulsation effect in the sigmoid sinus resulting in instantaneous resolution of the pulsatile tinnitus.

We have adopted the THI[16] in our study population as it is a brief, easily administered, and psychometrically robust measure that evaluates the impact of tinnitus on daily living. Quartiles calculated from raw scores were used to create a matrix of values representing tinnitus severity in five grades (1–5). Specifically, the test-retest reliability/repeatability was high. The 95 percent confidence interval for the THI was 20-points, indicating that a 20-point or greater change had to occur from test to retest for a change to be considered statistically significant at the 5 percent confidence level[28], thus minimizing the intra-observer bias. Further the use of THI in the evaluation of subjective venous tinnitus has been previously validated[29].

Minimal or no tinnitus are grouped together under grade-1 of THI, which warrants caution while reporting patients with “minimal tinnitus” separate from “no tinnitus”. All our patients had grade-1on the THI scale following venous stenting. However, 97% of them had “no tinnitus” while only one patient had persistent “minimal tinnitus” of unchanged severity (patient-9 on Fig 4), which is an important distinction. Review of the THI sum score rather THI grade can overcome this limitation (Fig 5).

We provide information regarding the pattern of stenosis and flow in the venous sinuses and laterality of pulsatile tinnitus. In our cohort, laterality of tinnitus was dependent on the side of venous sinus stenosis and dominance pattern of the dural venous system. In other words, unilateral pulsatile tinnitus is significantly associated with ipsilateral stenosis in a dominant venous sinus, and bilateral pulsatile tinnitus is associated with bilateral stenosis of a co-dominant transverse sinus system. Our findings are in agreement with published literature[13,30].

Headache and visual symptoms are the usual initial presenting features with variable association of pulsatile tinnitus in patients with IIH.[31,32] Pulsatile tinnitus was reported in 55–60% of the patients[33] and may even be its initial manifestation[15,17]. In our cohort, 78% of the patients complain of pulsatile tinnitus. This was less frequent after headaches (100%) and significantly more frequently than visual symptoms. Our findings support the published evidence that otologic symptoms can be the initial clinical manifestation in IIH even in the absence of papilledema or vision changes. Considering that IIH is the most common cause of pulsatile tinnitus,[17,34] the close association of IIH with dural venous sinus stenosis[34,35] and the potential initial presentation of IIH with pulsatile tinnitus even in the absence of visual symptoms, patients presenting with pulsatile tinnitus and venous sinus stenosis should be cautiously evaluated for underlying IIH in appropriate clinical context.

The frequency of a new stenosis in our series (3/29, 10.3%) was similar to that of Ahmed et al (6/52, 11.5%)[20]. Similar to their experience, there was venous hypertension across the new stenosis in our patients, along with recurrent pulsatile tinnitus. The tinnitus resolved immediately post-stenting in the two patients that were treated with stenting of the recurrent stenosis, replicating the result of initial stenting. This phenomenon lends further credence to the theory that the tinnitus in IIH patients with venous sinus stenosis is caused primarily by the stenosis and resolves immediately after stenting.

Shargorodsky et.al reported increased incidence of pulsatile tinnitus in obese patients as compared to those with a normal BMI[36]. With 97% of the study population having above-normal BMI, our results are concordant with published literature. However, BMI subgroup analysis showed no significant correlation between the BMI versus and severity of tinnitus in our study population.

### Limitations

Certainly, there is a subjective component and possible bias when using a self-rating scale for tinnitus, although frequently reported and widely used previously, have been validated for discriminative purposes only in clinical practice. Otologic evaluation based on pure tone audiometry is not included in this study. This precluded the objective evaluation of hearing impairment, which is an important aspect in tinnitus patients in general. However, as the patients reported complete resolution of tinnitus post-stenting as opposed to mere improvement or partial resolution, it’s reasonable to consider this change an actual effect rather than variability of the self-reported scale. Also, our study population includes only patients with proven IIH. These results must be applied with caution when extrapolated to non-IIH patients with pulsatile tinnitus.

## Conclusion

In our experience, venous sinus stenting is a feasible, safe and effective technique for successful treatment of pulsatile tinnitus in patients with IIH and significant venous sinus stenosis. Complete resolution of the tinnitus was demonstrated immediately after venous sinus stenting in our patient cohort. Larger prospective trials with specific focus on audiometric evaluation and long-term follow-up are necessary to confirm these findings. With this in mind, our group has initiated a prospective trial evaluating the safety and effectiveness of venous sinus stenting in patients with pulsatile tinnitus and venous sinus stenosis (ClinicalTrials.gov, NCT02734576).

## Supporting Information

S1 Dataset Study Data—Spread sheet of the anonymized study data. (XLSX)

## Abbreviations

IIH: Idiopathic Intracranial Hypertension
THI: Tinnitus Handicap Inventory
BMI: Body-mass index
MRV: Magnetic resonance venography
VSS: Venous sinus stenting
